# Insights Into the Inhibition of MOX-1 β-Lactamase by S02030, a Boronic Acid Transition State Inhibitor

**DOI:** 10.3389/fmicb.2021.720036

**Published:** 2021-12-14

**Authors:** Tatsuya Ishikawa, Nayuta Furukawa, Emilia Caselli, Fabio Prati, Magdalena A. Taracila, Christopher R. Bethel, Yoshikazu Ishii, Akiko Shimizu-Ibuka, Robert A. Bonomo

**Affiliations:** ^1^Department of Applied Life Sciences, Niigata University of Pharmacy and Applied Life Sciences, Niigata, Japan; ^2^Kagoshima Prefectural College, Kagoshima, Japan; ^3^Department of Life Sciences, University of Modena and Reggio Emilia, Modena, Italy; ^4^Louis Stokes Cleveland Department of Veterans Affairs Medical Center, Cleveland, OH, United States; ^5^Department of Medicine, Case Western Reserve University School of Medicine, Cleveland, OH, United States; ^6^Department of Microbiology and Infectious Diseases, Toho University School of Medicine, Tokyo, Japan; ^7^Departments of Pharmacology, Molecular Biology and Microbiology, Biochemistry, and Proteomics and Bioinformatics, Case Western Reserve University School of Medicine, Cleveland, OH, United States; ^8^CWRU-Cleveland VAMC Center for Antimicrobial Resistance and Epidemiology (Case VA CARES), Cleveland, OH, United States

**Keywords:** β-lactamase, BATSI, extended-spectrum AmpC, Ω-loop, β-lactamase inhibitor

## Abstract

The rise of multidrug resistant (MDR) Gram-negative bacteria has accelerated the development of novel inhibitors of class A and C β-lactamases. Presently, the search for novel compounds with new mechanisms of action is a clinical and scientific priority. To this end, we determined the 2.13-Å resolution crystal structure of S02030, a boronic acid transition state inhibitor (BATSI), bound to MOX-1 β-lactamase, a plasmid-borne, expanded-spectrum AmpC β-lactamase (ESAC) and compared this to the previously reported aztreonam (ATM)-bound MOX-1 structure. Superposition of these two complexes shows that S02030 binds in the active-site cavity more deeply than ATM. In contrast, the SO_3_ interactions and the positional change of the β-strand amino acids from Lys315 to Asn320 were more prominent in the ATM-bound structure. MICs were performed using a fixed concentration of S02030 (4 μg/ml) as a proof of principle. Microbiological evaluation against a laboratory strain of *Escherichia coli* expressing MOX-1 revealed that MICs against ceftazidime are reduced from 2.0 to 0.12 μg/ml when S02030 is added at a concentration of 4 μg/ml. The IC_50_ and *K*_i_ of S02030 vs. MOX-1 were 1.25 ± 0.34 and 0.56 ± 0.03 μM, respectively. Monobactams such as ATM can serve as informative templates for design of mechanism-based inhibitors such as S02030 against ESAC β-lactamases.

## Introduction

The most widespread mechanism of resistance to β-lactam antibiotics among Gram-negative bacteria is the production of β-lactamases (EC 3.5.2.6) (Wilke et al., 2005). Based on their primary sequences, β-lactamases are grouped into four major classes, A–D (Ambler, 1980; Jaurin and Grundstrom, 1981; Ouellette et al., 1987). Class A, C, and D β-lactamases possess a conserved catalytic serine residue in their active site; class B harbors one or two zinc metal ions. Class C β-lactamases are widely distributed among many bacteria and show increased catalytic efficiency toward cephalosporins, and are poorly inhibited by the mechanism-based β-lactamase inhibitors (BLIs) clavulanic acid, sulbactam, and tazobactam (Bush et al., 1995; Helfand and Bonomo, 2003). New β-lactamase inhibitors recently developed are highly effective against a broader range of β-lactamases than clavulanic acid and tazobactam and have particular activity against class C enzymes. These new inhibitors are from the diazabicyclooctanone (DBO) and boronate chemical families and both possess a β-lactam-like mode of action.

**FIGURE 1:**
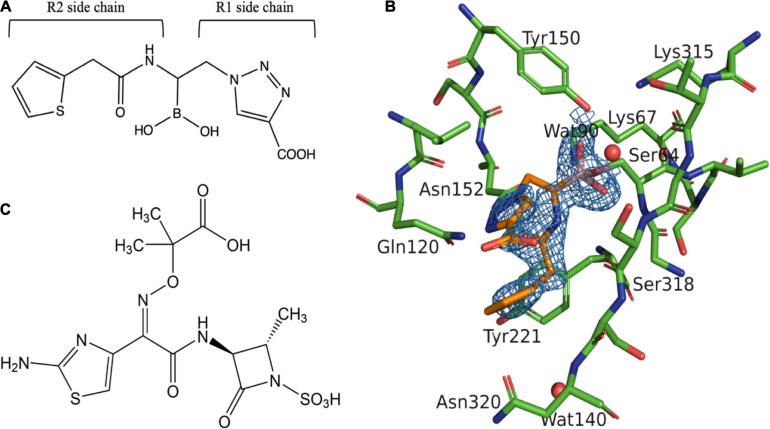
Structure of aztreonam (ATM) and S02030 bound to MOX-1 β-lactamase. **(A)** Chemical structure of S02030. **(B)** Electron density of S02030 bound to the active site of MOX-1. **(C)** Chemical structure of ATM.

Boronic acid transition state analog inhibitors (BATSIs) are undergoing rapid development (Docquier and Mangani, 2018). S02030, a BATSI (Figure 1A), was found to inhibit a broad range of β-lactamases both in class A and class C. Crystal structures of enzymes such as a class C β-lactamase ADC-7, class A enzymes SHV-1 and KPC-2 bound to S02030 have been reported (Powers et al., 2014; Nguyen et al., 2016). MOX-1 is a plasmid-mediated, CMY-type class C β-lactamase isolated from *Klebsiella pneumoniae* NU2936, a strain that is resistant to expanded-spectrum cephalosporins (Horii et al., 1993). Kinetic studies demonstrate that benzylpenicillin, cephalothin, cefcapene, and moxalactam are good substrates of MOX-1, while ceftazidime and cefepime are poor substrates and aztreonam (ATM; Figure 1C) behaves as an inhibitor (Alba et al., 2003).

The crystal structures of free and ATM-bound MOX-1 have been determined (Oguri et al., 2014, 2015). In both analyses, electron density for some amino acids in the Ω-loop (Asn212-Pro213) and N-terminus of B3 β-strand (from Glu306 to Ser309) were not observed, suggesting significant flexibility in these regions. The structure of ATM-bound MOX-1 (ATM: MOX-1) also revealed that the binding of ATM causes conformational change and structural disturbance of the B3 β-strand main chain (Oguri et al., 2015). In this present study, we determined the 2.13-Å resolution crystal structure of S02030 bound to MOX-1 β-lactamase. Difference of binding mode between S02030 and ATM was analyzed, and the correlation between the binding mode and inhibitory activity was investigated. Our goal was to exploit similarities and differences in binding between ATM and S02030 in order to inform better inhibitor design. We also performed comparative analysis of the S02030 binding mode in several structure-solved complexes. Based on this analysis, four new derivatives of S02030 were proposed for further investigation. The binding pattern of the new designed inhibitors was determined using *in silico* studies, by docking them into the active site of MOX-1 enzyme. In order to facilitate comparison, the amino acid residues of MOX-1 are designated with the standard numbering for class C β-lactamases as previously reported (Mack et al., 2020).

## Materials and Methods

### Preparation of MOX-1

Production and purification of MOX-1 with (His)_8_ tag at its N-terminus were performed as described previously (Oguri et al., 2014). For kinetic assays, the purified protein was treated with thrombin to remove the (His)_8_ tag. The sample was further purified with Hi-Trap Benzamidine FF column and His-Trap FF column (Cytiva).

### Kinetic Analysis

S02030 was chemically synthesized as previously described (Powers et al., 2014). Cephalothin (TIN) (Δε_262_ = −7,660 M^–1^ cm^–1^) was purchased from Sigma-Aldrich and used as substrate. ATM was purchased from Tokyo Chemical Industry Co., Ltd., MOX-1 was pre-incubated with inhibitor (S02030 or ATM) in assay buffer for 10 min before initiation of the reaction. The reaction was performed in 50 mM MOPS buffer (pH 7.0) containing 200 mM NaCl at 30°C with Jasco V-530 UV/VIS spectrophotometer. *K*_i_ and IC_50_ were determined as previously described (Drawz et al., 2011; Caselli et al., 2015).

### Minimal Inhibitory Concentration Assays

*Escherichia coli* BL21(DE3) cell (New England Biolabs) transformed with pET-28a plasmid harboring the MOX-1 gene was used for susceptibility assays. Minimal inhibitory concentration (MIC) assays were performed by a broth microdilution method following the guidelines of the Clinical and Laboratory Standards Institutes (CLSI, 2012). An inoculum of 10^4^ *E. coli* cells was grown in Mueller-Hinton broth at 37°C for 20 h in the presence of ATM with or without S02030 (4 μg/ml), supplied in serial dilutions in a 96-well microplate. *E. coli* BL21(DE3) cells transformed with the pET-28a plasmid without the MOX-1 gene served as negative control.

### Crystal Preparation, Data Collection, and Structure Determination

The purified (His)_8_-tagged MOX-1 was concentrated to 10 mg/mL with Amicon Ultra-4 10 K (Merck Millipore Ltd.), and it was subjected to crystallization. Crystals for X-ray analysis were grown at 16°C by the hanging-drop vapor diffusion method with reservoir solutions of 14–20% polyethylene glycol 8,000 in 100 mM sodium cacodylate buffer (pH 6.5) with 0.2 M zinc acetate. Crystals were soaked with S02030 by dipping in a reservoir solution containing 5–10 mM S02030 for 10–60 min. These crystals were flash frozen in a liquid nitrogen stream (100°K) with 20% ethylene glycol as a cryoprotectant and immediately subjected to data collection at Station 5A of the Photon Factory, the High Energy Accelerator Research Organization (KEK, Ibaraki, Japan). The reflection was indexed and integrated using iMosflm and SCALA in CCP4 package (Collaborative Computational Project Number 4, 1994). The initial model for molecular replacement was the structure of free MOX-1 (PDB entry: 3W8K). Molecular replacement and refinement were performed with MOLREP in CCP4 (Vagin and Teplyakov, 2010). The coordinate and structure factor files have been deposited in the Protein Data Bank under the accession code 5ZYB.

### Molecular Docking

The crystal structure of MOX-1 (PDB: 3W8K) was used to create the models. The structure was minimized using Discovery Studio (BIOVIA, 2020) molecular modeling software (BIOVIA, 2020). The water molecules were deleted, and the new compounds were built and minimized. CDOCKER (Wu et al., 2003) protocol was used to dock the new compounds into the active site of MOX-1 and analyzed.

## Results and Discussion

### Inhibition Kinetics and Microbiological Data (MIC)

In order to assess the affinity of the studied ligands, we determined both the *K*_i_ and IC_50_. S02030 and ATM were incubated with MOX-1 for 10 min and velocities were determined. The IC_50_ and *K*_i_ values for S02030 were 1.25 ± 0.34 μM and 0.563 ± 0.030 μM, respectively. These values are greater than those for ATM (Table 1) and suggest that ATM is a more potent inhibitor with higher affinity to MOX-1 enzyme than S02030.

**TABLE 1:** IC50 and Ki values of S02030 and aztreonam.

Inhibitor	IC_50_ (μM)	*K*_*i*_ (μM)
S02030	1.25 ± 0.34	0.56 ± 0.03
Aztreonam	0.02 ± 0.01	0.010 ± 0.001

To assess the microbiological potency of S02030 compared to ATM, we determined MICs in a uniform genetic background (Table 2). Against *E. coli* BL21 (DE3) expressing MOX-1, the MICs of ATM are 1 μg/ml. With 4 μg/ml of S02030, MICs were reduced to 0.125 μg/ml. With regards to cefoxitin (FOX), MICs were reduced from 32 to 8 μg/ml using the same concentration of S02030. For ceftazidime (CAZ), MICs were reduced by four dilutions, from 2 to 0.125 μg/ml.

**TABLE 2:** MIC data of antibiotics* in presence and absence of S02030 (4 μg/ml).

	ATM	ATM + S02030	FOX	FOX + S02030	CAZ	CAZ + S02030
*E. coli* BL21(DE3) *bla*_MOX–1_/pET-28a	1	0.125	32	8	2	0.12

*Aztreonam (ATM), cefoxitin (FOX), and ceftazidime (CAZ).

### Crystal Structure

The crystal structure of S02030-bound MOX-1 (S02030: MOX-1) was determined at 2.13-Å resolution with *R* and *R*_free_ factors of 15.8 and 20.7%, respectively (Table 3). Space group and cell parameters are basically identical with the previous data for free and ATM-bound MOX-1, and there is one molecule in an asymmetric unit.

**TABLE 3:** Data collection and refinement statistics.

**Data collection**	
Space group	*P*2_1_
Cell parameters (Å, deg)	*a* = 49.64, *b* = 59.20, *c* = 62.89 α = γ = 90.00, β = 102.03
Resolution (Å)	2.13–61.51 (2.13–2.25)
Observed reflections	63550 (9337)
Unique reflections	20024 (2908)
Completeness (%)	99.9 (100.0)
Redundancy	3.2 (3.2)
*R*_merge_	0.079 (0.188)
*I*/σ (I)	10.8 (5.5)
**Refinement**	
Resolution range for refinement (Å)	2.13–42.69 (2.13–2.18)
Unique reflections	19050 (1397)
Completeness (%)	99.84 (100.00)
Number of atoms	2881
Protein	2631
Water	190
Other atoms	60
*R*_work_	0.158 (0.173)
*R*_free_	0.205 (0.231)
**Average B factor (Å^2^)**	
All atoms	24.88
Protein	24.13
Ligand (ZXM)	56.31
Water	29.82
Other solvent molecules	24.54
**Root mean square deviation**	
Bond lengths (Å)	0.009
Bond angles (deg)	1.279

The final S02030: MOX-1 model includes amino acid residues from Asp6 to Arg305 and from Gln310 to Gly362. The overall structure is almost identical to that of free MOX-1 (PDB accession code: 3W8K), with a root-mean-square deviation (RMSD) of 0.099 Å for all atoms. Electron density for the N-terminus of B3 β-strand (from Glu306 to Ser309) was not observed as in the case of previous structural analyses of MOX-1. On the other hand, Asn213-Pro214, a part of Ω loop, which was unclear in the previous structural analyses of free and ATM-bound MOX-1, is observed in this S02030-bound structure.

### Binding Mode of S02030

**FIGURE 2:**
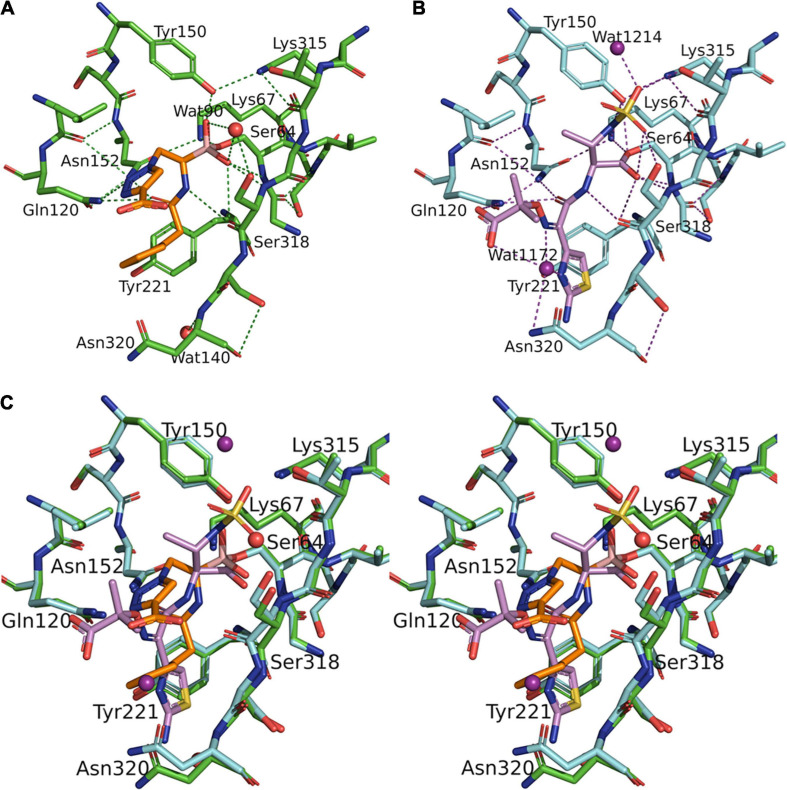
Structural comparison between S02030: MOX-1 and ATM: MOX-1. Hydrogen-bonding pattern between S02030 and MOX-1 (A) and between ATM and MOX-1 (B). (C) Superposition of active site structures of S02030: MOX-1 (orange: green) and ATM: MOX-1 (violet: cyan).

In S02030: MOX-1 structure, the temperature factor for S02030 is high, and the electron density for solvent exposed tail of R2 side chain is not clearly observed (Table 3 and Figure 1B). The S02030 boron atom is covalently attached to the catalytic Ser64 residue, and there are several hydrogen bonds between the inhibitor and the enzyme (Figure 2A). The oxygen atom of the borate hydroxyl group is positioned in the oxyanion hole and makes hydrogen bonds with the main chain nitrogen and oxygen atoms of Ser318, and nitrogen atom of Ser64. The oxygen atom of the other hydroxyl group is hydrogen-bonded to the side-chain oxygen atom of Tyr150. The nitrogen atom of the R1 amide group interacts with the main chain oxygen atom of Ser318, and the oxygen of the same group interacts with the side chain nitrogen atoms of Gln120 and Asn152.

### Structural Comparison Between S02030 and Aztreonam Bound to MOX-1

The structure of MOX-1 in S02030-bound MOX-1 (S02030: MOX-1) is similar to that in ATM-bound MOX-1 (ATM: MOX-1, and PDB entry code 4WBG), with a RMSD or 0.113 Å for all atoms. Comparison of hydrogen bonding pattern in these two complexes suggests that ATM is more tightly bound to the active site of the enzyme through its SO_3_ group, since several hydrogen bonds are formed between this group and the active-site residues of MOX-1 (Figure 2B), and electron density for this SO_3_ group was fairly high (Oguri et al., 2015). Structural superposition of these two structures indicates that S02030 binds to the active-site cavity more deeply than ATM (Figure 2C). Binding of ATM effected a conformational change and/or structural disturbance in the β-strand amino acids from Lys315 to Asn320, with significantly high B-factor values in their Cα atoms (Oguri et al., 2015). The binding of S02030 does not impact the position of these residues, with no elevation of B-factor values in this region.

### Comparison of S02030 Binding With Other β-Lactamases

**FIGURE 3:**
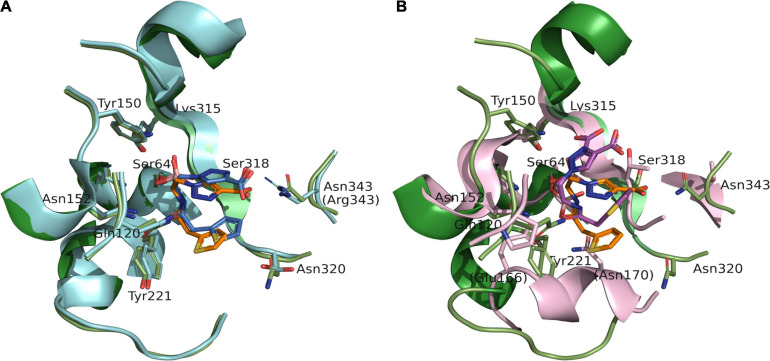
Superposition of S02030: MOX-1 (orange: green) and previously analyzed crystal structures of S02030: ADC-7 (PDB: 4U0X) [(A) blue: cyan] and S02030: KPC-2 (PDB: 5EEC) [(B) violet: pink]. The labels in parentheses indicate the residues of ADC-7 (A) and KPC-2 (B).

The binding position of S02030 to the active-site cavity of MOX-1 was compared with that of S02030-bound class C β-lactamase ADC-7 (PDB entry code: 4U0X) (Figure 3A; Powers et al., 2014). Four independent molecules determined in the crystallographic analysis of S02030-bound ADC-7 showed some variety in the position of S02030, and here we compared our S02030: MOX-1 structure with the C monomer.

The binding mode is very similar in both enzymes, except the position of R2 side chain. In the S02030: ADC-7 complex, the carboxylate group of R2 group interacts with Arg343. The position of this Arg is occupied with Asn in MOX-1, and lack of the interaction should cause the difference in the position and flexibility of R2 side chain between these two enzymes. This is in agreement with the fact that the electron density for the R2 side chain is less clear in S02030: MOX-1 than S02030: ADC-7, and with the *K*_i_ values of S02030 for these two enzymes: *K*_i_ for MOX-1 was 563 ± 30 nM, while that for ADC-7 was reported to be 44.5 nM. It was also reported that SM23, an inhibitor with shorter R2 group which allow to form more favorable interaction with Arg343, has higher affinity for ADC-7 than S02030 (Powers et al., 2014).

S02030 binds to the active site in a shallower position in class A β-lactamase KPC-2 (PDB entry code: 5EEC) (Figure 3B). This is due to the structural difference that class A KPC-2 has its Ω loop of helical structure that narrows the binding site for R1 group. Binding of boron oxygen with Glu166 and Asn170 in class A enzymes also changes the orientation of amide chain of S02030 R1 group (Nguyen et al., 2016).

**FIGURE 4:**
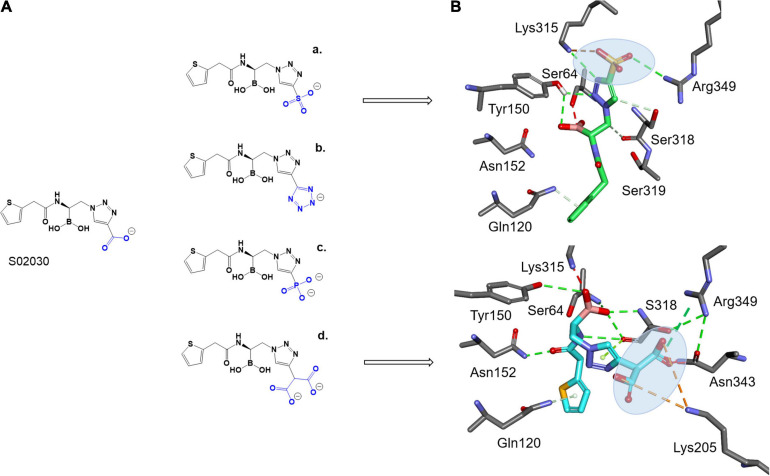
Proposed derivatives of S02030 that mimic ATM (A) docked as acyl enzyme complexes into the active site of MOX-1 (B). The new added moieties are targeting the active site residues Lys315, Asn343, and new interactions with Arg349 and Lys205 for better inhibition.

To take advantage of the possible interaction of inhibitors with Lys315, as seen with the ATM interaction and Arg346 (present in both ADC-7 and MOX-1), and shorter side chain of Asn343 vs. Arg343 in ADC-7, the carboxyl group of S02030 was replaced by a series of negatively charged groups (Figure 4A). The new compounds with sulfate group SO_3_ (a), tetrazole (b), phosphate (c), and double carboxyl (d) were docked into the active site of MOX-1; the results show that the SO_3_ group (Figure 4B) makes interactions with Lys315 and Arg346. Interestingly, the double carboxyl group additionally forms interactions not only with Asn343, Arg346, but also with Lys205 (Gln205 in ADC-7). The interactions with Gln120 and Asn152 were preserved, as well as the interactions of boronic groups with Tyr150 and Ser318.

## Conclusion

Inhibition of extended-spectrum β-lactamases is a continuing challenge. In class C β-lactamase MOX-1, the B3 β-strand amino acids from Lys315 to Asn320 change position when complexed with ATM in contrast to S02030, a BATSI effective both for class A and class C enzymes. S02030, as a transition state mimic, binds more deeply in the active site and does not impact a conformational change. Hydrogen-bonding interaction between inhibitors and MOX-1 shows that ATM is more tightly bound to the active site through its SO_3_ group, a feature missing in S02030. Such structural features are consistent with the result of kinetic analysis that showed that the IC_50_ and *K*_i_ values for S02030 are greater than those for ATM, indicating that S02030 behaves as a less potent inhibitor than ATM. These observations suggest that a novel approach forward in BATSI design would be to add an SO_3_ group in the region of the R1 side chain to effect greater hydrogen binding opportunities.

## Data Availability Statement

The datasets presented in this study can be found in online repositories. The names of the repository/repositories and accession number(s) can be found in the article/supplementary material.

## Author Contributions

TI and NF performed the kinetic and X-ray crystallographic analysis. EC and FP designed and synthesized S02030. MT performed the MICs and molecular modeling. RAB conceived of the experiment, directed research, and contributed to wrote the manuscript. YI performed the MIC investigation and validation, and contributed to writing the manuscript. AS-I and CB contributed to writing the manuscript. All authors contributed to the article and approved the submitted version.

## Author Disclaimer

The content is solely the responsibility of the authors and does not necessarily represent the official views of the NIH or the Department of Veterans Affairs.

## Conflict of Interest

The authors declare that the research was conducted in the absence of any commercial or financial relationships that could be construed as a potential conflict of interest.

## Publisher’s Note

All claims expressed in this article are solely those of the authors and do not necessarily represent those of their affiliated organizations, or those of the publisher, the editors and the reviewers. Any product that may be evaluated in this article, or claim that may be made by its manufacturer, is not guaranteed or endorsed by the publisher.

## References

[B1] Alba J. Bauvois C. Ishii Y. Galleni M. Masuda K. Ishiguro M. (2003). A detailed kinetic study of Mox-1, a plasmid-encoded class C β-lactamase. *FEMS Microbiol. Lett.* 225 183–188. 10.1016/S0378-1097(03)00448-8 12951239

[B2] Ambler R. P. (1980). The structure of β-lactamases. *Philos. Trans. R. Soc. Lond. B Biol. Sci.* 289 321–331. 6109327 10.1098/rstb.1980.0049

[B3] BIOVIA (2020). *Discovery Studio 2020 Client, 3DEXPERIENCER Platform.* San Diego, CA: Dassault Systèmes. Available online at: https://www.3ds.com/biovia

[B4] Bush K. Jacoby G. A. Medeiros A. A. (1995). A functional classification scheme for β-lactamases and its correlation with molecular structure. *Antimicrob. Agents Chemother.* 39 1211–1233. 10.1128/AAC.39.6.1211 7574506 PMC162717

[B5] Caselli E. Romagnoli C. Vahabi R. Taracila M. A. Bonomo R. A. Prati F. (2015). Click chemistry in lead optimization of boronic acids as beta-lactamase inhibitors. *J. Med. Chem.* 58 5445–5458. 10.1021/acs.jmedchem.5b00341 26102369 PMC5744665

[B6] CLSI (2012). *Methods for Dilution Antimicrobial Susceptibility Tests for Bacteria That Grow Aerobically; Approved Standard*, 9th Edn. Wayne, PA: Clinical and Laboratory Standards Institute.

[B7] Collaborative Computational Project Number 4 (1994). The CCP4 suite: programs for protein crystallography. *Acta Crystallogr. D Biol. Crystallogr.* 50 760–763. 15299374 10.1107/S0907444994003112

[B8] Docquier J. D. Mangani S. (2018). An update on beta-lactamase inhibitor discovery and development. *Drug. Resist. Updat.* 36 13–29. 29499835 10.1016/j.drup.2017.11.002

[B9] Drawz S. M. Taracila M. Caselli E. Prati F. Bonomo R. A. (2011). Exploring sequence requirements for C(3)/C(4) carboxylate recognition in the *Pseudomonas aeruginosa* cephalosporinase: insights into plasticity of the AmpC beta-lactamase. *Protein Sci.* 20 941–958. 10.1002/pro.612 21404358 PMC3104225

[B10] Helfand M. S. Bonomo R. A. (2003). Beta-lactamases: a survey of protein diversity. *Curr. Drug Targets Infect. Disord.* 3 9–23. 12570729 10.2174/1568005033342181

[B11] Horii T. Arakawa Y. Ohta M. Ichiyama S. Wacharotayankun R. Kato N. (1993). Plasmid-mediated AmpC-type beta-lactamase isolated from *Klebsiella pneumoniae* confers resistance to broad-spectrum beta-lactams, including moxalactam. *Antimicrob. Agents Chemother.* 37 984–990. 10.1128/AAC.37.5.984 8517725 PMC187871

[B12] Jaurin B. Grundstrom T. (1981). ampC cephalosporinase of *Escherichia coli* K-12 has a different evolutionary origin from that of beta-lactamases of the penicillinase type. *Proc. Natl. Acad. Sci. U.S.A.* 78 4897–4901. 10.1073/pnas.78.8.4897 6795623 PMC320287

[B13] Mack A. R. Barnes M. D. Taracila M. A. Hujer A. M. Hujer K. M. Cabot G. (2020). A standard numbering scheme for Class C beta-Lactamases. *Antimicrob. Agents Chemother.* 64:e02247-19. 10.1128/AAC.01841-19 31712217 PMC7038296

[B14] Nguyen N. Q. Krishnan N. P. Rojas L. J. Prati F. Caselli E. Romagnoli C. (2016). Crystal structures of KPC-2 and SHV-1 beta-Lactamases in complex with the boronic acid transition state analog S02030. *Antimicrob. Agents Chemother.* 60 1760–1766. 10.1128/AAC.02643-15 26729491 PMC4775972

[B15] Oguri T. Furuyama T. Okuno T. Ishii Y. Tateda K. Bonomo R. A. (2014). Crystal structure of Mox-1, a unique plasmid-mediated Class C beta-Lactamase with hydrolytic activity towards moxalactam. *Antimicrob. Agents Chemother.* 58 3914–3920. 10.1128/AAC.02363-13 24777102 PMC4068568

[B16] Oguri T. Ishii Y. Shimizu-Ibuka A. (2015). Conformational change observed in the active site of Class C beta-Lactamase MOX-1 upon binding to aztreonam. *Antimicrob. Agents Chemother.* 59 5069–5072. 10.1128/AAC.04428-14 26055361 PMC4505233

[B17] Ouellette M. Bissonnette L. Roy P. H. (1987). Precise insertion of antibiotic resistance determinants into Tn21-like transposons: nucleotide sequence of the OXA-1 beta-lactamase gene. *Proc. Natl. Acad. Sci. U.S.A.* 84 7378–7382. 2823258 10.1073/pnas.84.21.7378 PMC299299

[B18] Powers R. A. Swanson H. C. Taracila M. A. Florek N. W. Romagnoli C. Caselli E. (2014). Biochemical and structural analysis of inhibitors targeting the ADC-7 cephalosporinase of *Acinetobacter baumannii*. *Biochemistry* 53 7670–7679. 10.1021/bi500887n 25380506 PMC4263437

[B19] Vagin A. Teplyakov A. (2010). Molecular replacement with MOLREP. *Acta Crystallogr. D Biol. Crystallogr.* 66 22–25. 20057045 10.1107/S0907444909042589

[B20] Wilke M. S. Lovering A. L. Strynadka N. C. (2005). β-Lactam antibiotic resistance: a current structural perspective. *Curr. Opin. Microbiol.* 8 525–533. 10.1016/j.mib.2005.08.016 16129657

[B21] Wu G. Robertson D. H. Brooks C. L. III Vieth M. (2003). Detailed analysis of grid-based molecular docking: a case study of CDOCKER-A CHARMm-based MD docking algorithm. *J. Comput. Chem.* 24 1549–1562. 10.1002/jcc.10306 12925999

